# The Effect of Climate Change on Indicator Wetland Insects: Predicting the Current and Future Distribution of Two Giant Water Bugs (Hemiptera: Belostomatidae) in South Korea

**DOI:** 10.3390/insects15100820

**Published:** 2024-10-19

**Authors:** Seon Yi Kim, Changseob Lim, Ji Hyoun Kang, Yeon Jae Bae

**Affiliations:** 1 Department of Life Sciences, Graduate School, Korea University, Seoul 02841, Republic of Korea; paimon@korea.kr; 2 Biodiversity Research Department, Species Diversity Research Division, National Institute of Biological Resources, Incheon 22689, Republic of Korea; 3 Korean Entomological Institute, Korea University, Seoul 02841, Republic of Korea; stagbeetle95@korea.ac.kr (C.L.); jihyounkang@korea.ac.kr (J.H.K.); 4 Ojeong Resilience Institute, Korea University, Seoul 02841, Republic of Korea; 5 Division of Environmental Science and Ecological Engineering, College of Life Sciences and Biotechnology, Korea University, Seoul 02841, Republic of Korea

**Keywords:** *Appasus japonicus*, aquatic insects, conservation, *Diplonychus esakii*, maximum entropy model, range shift

## Abstract

**Simple Summary:**

Giant water bugs play an important role as top predators in wetland ecosystems, helping to control freshwater snails and mosquitoes. In this study, we used MaxEnt models to predict how climate change might affect the distribution of two Korean species, *Appasus japonicus* and *Diplonychus esakii*. Our findings suggest that *A. japonicus* may lose habitat and shift northward, while *D. esakii* is expected to expand its range, potentially causing occupancy turnover between the two species. The range shifts of the two species are primarily driven by different factors—elevation for *A. japonicus* and annual mean temperature for *D. esakii*. This research helps us understand how climate change could affect two giant water bugs and supports efforts to manage and conserve wetland ecosystems in South Korea.

**Abstract:**

Giant water bugs (Hemiptera: Belostomatidae) are top predators in wetland ecosystems, serving as biological indicators of the health of lentic ecosystems and as effective biological control agents for freshwater snails and mosquitoes. This study aimed to predict the current and future distribution of two Korean giant water bugs, *Appasus japonicus* and *Diplonychus esakii*, under three climate change scenarios, contributing to the sustainable management of wetland ecosystems in South Korea. Using MaxEnt models, we employed seven climatic and three non-climatic variables to investigate the habitat preferences and distribution patterns of the species. The results revealed that *A. japonicus* is likely to experience a northward range contraction due to climate change, while *D. esakii* is predicted to expand its distribution northward without losing its current range. These responses may lead to occupancy turnover between the two species, potentially driving reassembly in aquatic organism community. Elevation was the primary factor influencing the distribution of *A. japonicus*, whereas annual mean temperature was the most informative variable for *D. esakii*, both factors derived under the current climate conditions. These findings suggest that both species are highly sensitive to climate change, with potential range shifts toward higher latitudes and elevations. This study provides insights into how climate change could impact two giant water bugs, thereby supporting future efforts to manage and conserve wetland ecosystems in this country.

## 1. Introduction

The giant water bug family Belostomatidae (Hemiptera: Heteroptera: Nepomorpha), known for having the largest body size among aquatic insects, contains species that can grow up to 110 mm in length [1,2]. To date, approximately 170 species have been recorded worldwide, with over 110 species found in tropical regions [3,4]. In the Palearctic region, 14 species of the family Belostomatidae have been recorded, including four species in the genus *Appasus* and three species in the genus *Diplonychus*, which are the focus of this study [5]. Additionally, four species of the Belostomatidae family—*Kirkaldyia deyrolli* (Vuillefroy, 1864), *Appasus japonicus* (Vuillefroy, 1864), *A. major* (Esaki, 1934), and *Diplonychus esakii* Miyamoto & Lee, 1966—have been recorded in South Korea [5,6,7]. These four giant water bug species in the Palearctic region exhibit different distribution patterns across China, Japan, Russia, Taiwan, and South Korea, with all four species recorded as being co-distributed in both South Korea and Japan [5].

As top predators in aquatic ecosystems, giant water bugs primarily inhabit lentic environments and feed on a wide range of aquatic organisms such as insects, cladocerans, amphipods, amphibians, and small fish [8,9,10]. They play an important role in wetland food webs as predators and are used as biological indicators of the health of lentic ecosystems [11]. Furthermore, they are recognized as effective biological control agents of agricultural pests and disease vectors such as freshwater snails and mosquitoes [11,12,13]. Some nepomorph (Hemiptera: Heteroptera: Nepomorpha) species, including the two giant water bug species examined in this study, prey on snails, which can potentially serve as intermediate hosts for human pathogens like schistosomes [14]. Notably, the genus *Diplonychus* is known to prey on mosquito larvae, making it a promising biological control agent for diseases like dengue fever and malaria in tropical regions [3,15].

Among the four Korean Belostomatidae, *K. deyrolli* is the largest species (50–70 mm) and is listed as a Class II endangered wildlife species in South Korea, with its natural habitats restricted to only a few islands, such as Jeju Island and Ganghwa Island [16,17]. The remaining three giant water bugs (*A. japonicus*, *A. major*, and *D. esakii*) are top predators that inhabit various lentic environments, such as wetlands, ponds, reservoirs, and small water bodies [3,18,19,20]. *Appasus japonicus* is widely distributed across South Korea and is frequently observed even in urban habitats. In contrast, a rare species, *A. major*, tends to inhabit relatively higher altitude areas than *A. japonicus* [21], but little is known about its distribution in South Korea. Furthermore, due to the morphological similarities between *A. major* and *A. japonicus* [22], accurate identification is often challenging, raising doubts about the reliability of occurrence records for *A. major*. *Diplonychus esakii* is primarily found in low-altitude areas of the southern regions and Jeju Island [23]. Therefore, this study was designed to focus on *A. japonicus* and *D. esakii*, which have well-documented distribution data, to investigate the potential distribution of Korean giant water bugs. *Kirkaldyia deyrolli*, already endangered with an extremely reduced range, and *A. major*, due to its insufficient data, were not included.

Climate change can lead to biodiversity losses across different ecological scales and, in particular, drive shifts in species’ distribution ranges. In recent decades, Species Distribution Models (SDMs) have been widely utilized to understand and predict species’ geographic distributions and habitat suitability by incorporating environmental variables and known occurrence records [24]. SDMs allow us to extrapolate models to different environmental layers, enabling predictions of how species distribution might change in different environmental conditions and time periods [24,25]. The maximum entropy model (MaxEnt) is one of the most popular SDMs, a machine learning approach that uses presence-only data to estimate species distribution [24,25,26]. MaxEnt predicts distributions by finding the maximum entropy distribution under current environmental constraints [27]. This model is particularly useful for species with limited occurrence records, making it beneficial for studying various organisms, including endangered insects [28,29,30].

Previous studies on the range shifts, abundance, and phenology changes of aquatic Heteroptera under climate change scenarios provide valuable insights relevant to the giant water bug species examined in this study. Research conducted in the UK and other parts of Europe comparing species distribution between 1970 and 1980 and 1990 and 2000 observed notable range expansions in aquatic Heteroptera, particularly with southern species extending their ranges northward and to higher altitudes [31,32]. Simulation models based on climate change scenarios predict an increase in the abundance of dominant species within aquatic Heteroptera communities, along with phenological shifts that may advance by 20 to 50 days, or in some cases, experience delays [33,34]. These studies highlight that Heteroptera species have expanded their ranges northward, a trend particularly pronounced in northern Europe.

Globally, inland and coastal wetlands cover approximately 12.1 million km^2^, but natural wetlands are in long-term decline [35]. Between 1970 and 2015, both inland and marine/coastal wetlands experienced a loss of about 35% of their area, a rate three times faster than that of forest loss. A quarter of wetland-dependent species are threatened with extinction, and since 1970, the population sizes of inland wetland species have declined by 81%, while the population sizes of marine and coastal wetland species have declined by 36% [35]. Major drivers of global wetland loss include urbanization, agricultural activities, climate change, and pollution, with agricultural activities having one of the most significant impacts [36].

In South Korea, giant water bugs used to be commonly found in shallow, small ponds near agricultural fields, but habitat loss due to urbanization and chemical pollution has led to a decline in their population sizes and distribution range [37]. The range shifts and declines of top predators like giant water bugs may cause reassembly in aquatic communities, highlighting the need for conservation efforts. Additionally, giant water bugs are sensitive to changes in temperature and humidity [38], and thus, their range shifts can provide important insights into the responses of aquatic insects to environmental changes. However, aside from *K. deyrolli*, the distribution and ecological niche of Korean Belostomatidae remain poorly understood.

Therefore, this study aims to predict the current and future distribution of two Korean giant water bug species under climate change, as well as to compare their habitat preferences, contributing to the sustainable management and conservation of wetland ecosystems in South Korea.

## 2. Materials and Methods

### 2.1. Occurrence Records

**Figure 1:**
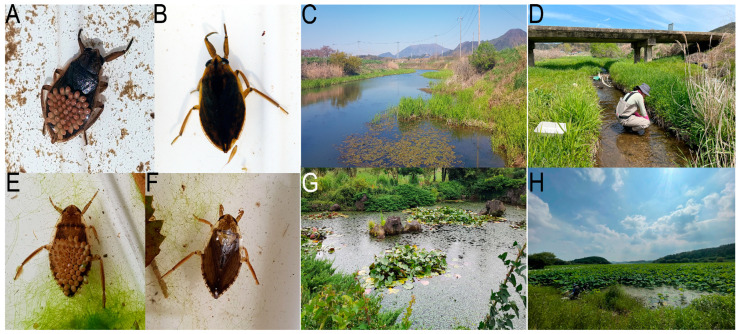
*Appasus japonicus* (**A**,**B**) and its habitats: agricultural wetland (**C**) and riverine wetland (**D**); *Diplonychus esakii* (**E**,**F**) and its habitats: artificial pond (**G**) and natural wetland (**H**). Photo by Seon Yi Kim (**A**–**C**), Min Jeong Baek * (**D**), Yeon Jae Bae (**E**–**G**), and Soo Ho Uh * (**H**). * National Institute of Biological Resources (Incheon, Republic of Korea).

Species occurrence records were gathered from three different sources in South Korea: (1) the Basic Survey on Inland Wetlands (2016 to 2021), downloaded from EcoBank [39]; (2) specimen metadata, downloaded from the Biodiversity Information System of the National Institute of Biological Resources [40]; and (3) specimens collected by the authors and colleagues in 2002 and 2023 (Figure 1). In total, 713 occurrence records for *A. japonicus* and 184 for *D. esakii* were obtained. To avoid duplicates and autocorrelated records, occurrence records within 1 km of each other were rarefied. After rarefaction, 671 (Table S1) and 154 (Table S2) occurrence records were retained, respectively, for subsequent species distribution modeling. The specimens were identified using the taxonomic key from Aquatic Insects of Korea [41] and the morphological distinctions between *A. japonicus* and *A. major* described by Suzuki et al. (2013) [22].

### 2.2. Environmental Variables

In this study, 19 climatic (bioclimatic variables BIO01–BIO19) and three non-climatic variables (altitude, distance from wetland, and land cover) were used for environmental variables (Table 1). Bioclimatic variables and the digital elevation model (DEM) were obtained at a spatial resolution of 30 arcsec (~1 km^2^) from Chelsa [42,43]. The distance from the wetland (DISTANCE) was calculated using the Korean “Wetland Inventory” shapefile from the National Institute of Ecology [44] (https://www.data.go.kr, accessed on 14 September 2024) and transformed into a 30 arcsec resolution layer [44]. Land cover data (LANDCOVER) were modified from Song et al. (2018), whose original layer had a 30 arcsec resolution [45]. Both the DISTANCE and LANDCOVER variables were transformed to the World Geodetic System (WGS1984) to ensure consistency with the other variables [46].

To prevent multicollinearity and overfitting of variables during modeling, a Pearson’s correlation analysis was performed using the SDM Toolbox v.2.0 [47] to eliminate highly correlated climatic variables (Pearson’s correlation coefficient >|0.8|). Among the highly correlated variables, we selected the most representative ones; for example, we retained BIO1 (annual mean temperature) while removing BIO6, BIO8, BIO9, and BIO10 for our analyses (Figure S1). As a result, seven climatic variables—annual mean temperature (BIO1), mean diurnal range (BIO2), maximum temperature of warmest month (BIO5), annual precipitation (BIO12), precipitation of wettest month (BIO13), precipitation of driest month (BIO14), and precipitation seasonality (BIO15)—were selected for species distribution modeling (Figure S1).

### 2.3. Climate Change Scenarios

The future distribution of the species under climate change was forecasted based on the three representative scenarios of Shared Socioeconomic Pathways (SSPs), estimated by MRI-ESM2-0 [48]: SSP126, SSP370, and SSP585. The first number in each scenario refers to the anticipated success or failure of future socio-economic conditions in mitigating and adapting to climate change, while the latter two numbers represent levels of radiative forcing per unit area, as defined in the previous Representative Concentration Pathways (RCPs) [49]. (e.g., In SSP126, the number 1 denotes the Sustainability pathway (SSP1), which emphasizes global cooperation, sustainable development, and environmental stewardship. The number 2.6 refers to RCP 2.6, indicating low radiative forcing (2.6 W/m^2^), aligned with strong mitigation efforts to limit global temperature rise to below 2 °C, ideally around 1.5 °C [49]).

SSP126 assumes a ‘green’ future, in which climate change mitigation and adaptation are successfully implemented, making it the most optimistic scenario of the three. SSP370 describes a scenario where both climate change adaptation and mitigation fail, leading to increased regional conflicts. SSP585 represents a failure in climate change mitigation, but continued fossil fuel-driven economic growth, resulting in accelerated climate change [29,50]. The future time periods of the 2055s and 2085s were represented by averaged layers for 2041–2070 and 2071–2100, respectively.

### 2.4. Species Distribution Modeling

Both current and future distributions of the two giant water bugs were inferred using maximum entropy modeling [51]. The “ENMeval” R package [52], implemented in R v.4.4.1 [53], was used to optimize two key parameters: the regularization multiplier (RM) and feature combination (FC). SDMs were generated through ten-fold cross-validation with 10,000 background points. The feature class includes Linear (L), Quadratic (Q), Product (P), Hinge (H), and Threshold (T). In this process, occurrence records were randomly split into ten subsets, with the model iterating over the same settings, using a single subset as the test data. RM values ranged from 0.5 to 4.0 in increments of 0.5, and six feature combinations (L, LQ, H, LQH, LQHP, and LQHPT) were tested, resulting in 48 parameter combinations of SDMs for each species.

The generated SDMs were evaluated using AUC_test_ value [25]. In maintaining a balance between predictive accuracy and model complexity, the values of AUC_diff_ and delta AICc [25,54] were also considered (Figure S2). To assess the relative importance of each variable, a Jackknife test was performed by comparing the regularized training gain. The percentage contribution and permutation importance were also calculated. The optimal SDMs were projected to three SSP scenarios in two time periods (2055s and 2085s). Presence–absence binary maps were created for each scenario using ten percentile thresholds to estimate future range shifts of each species (i.e., contraction, retention, and expansion). All SDMs were generated using MaxEnt v.3.4.4 and ENMeval R package [51,52].

## 3. Results

### 3.1. Current Distribution of Two Korean Giant Water Bugs

The optimal parameter settings for *A. japonicus* and *D. esakii* were RM values of 0.5 and 2, respectively, with both models applying the LQHP as a feature combination (Figure S2). The optimal model for each species demonstrated reliable AUC values, with *A. japonicus* showing a mean Training AUC of 0.792 and a mean Test AUC of 0.775, while *D. esakii* had a mean Training AUC of 0.901 and a mean Test AUC of 0.889, both indicating good model performance [55].

**Figure 2:**
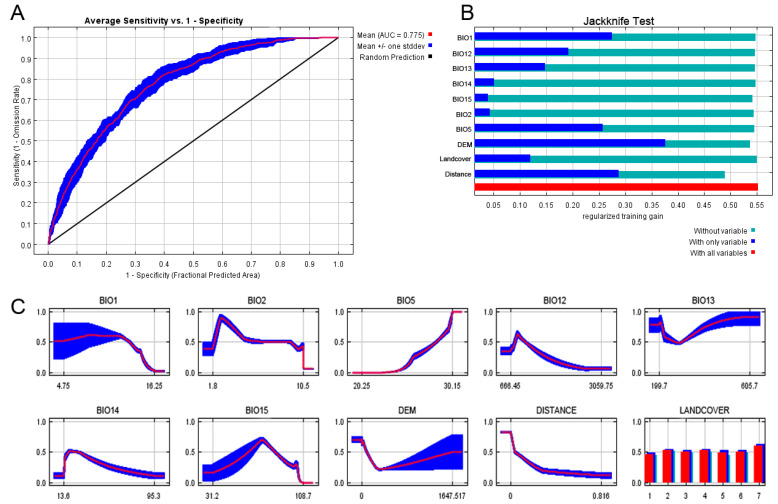
Area under the receiver operating curve (AUC) (**A**), jackknife test (**B**), and response curves of variables (**C**) of the optimal MaxEnt model for *Appasus japonicus*. (**A**) The red line represents the mean value across ten replicates and the blue shaded area indicates the standard deviation. (**B**) Relative information gain is depicted by blue, teal, and red bars, corresponding to the isolation, exclusion, and full models, respectively. (**C**) Response curves for each variable (Figure S3). Numbers on the *x*-axis of LANDCOVER indicate different classes: 1, urban; 2, agricultural; 3, forest; 4, grasslands; 5, wetland; 6, barren; and 7, water.

**Figure 3:**
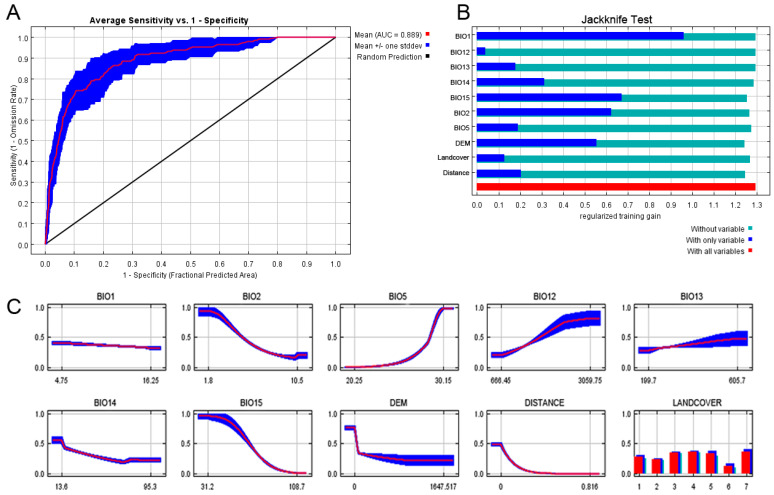
Area under the receiver operating curve (AUC) (**A**), jackknife test (**B**), and response curves (**C**) of the optimal MaxEnt model for *Diplonychus esakii*. (**A**) The red line represents the mean value among ten replicates and the blue shaded area indicates the standard deviation. (**B**) Relative information gain is depicted by blue, teal, and red bars, corresponding to the isolation, exclusion, and full models, respectively. (**C**) Response curves for each variable (Figure S4). Numbers on the *x*-axis of LANDCOVER indicate different classes: 1, urban; 2, agricultural; 3, forest; 4, grasslands; 5, wetland; 6, barren; and 7, water.

In the Jackknife test for *A. japonicus*, DEM (Surface elevation above sea level) provided the highest information gain when used alone (Figure 2). The response curve for DEM showed a sharp decline within the 0–300 m elevation range, suggesting a higher likelihood of occurrence in lowland areas. However, the standard deviation increased beyond the 300 m range. For *D. esakii*, the Jackknife test indicated that BIO1 (annual mean temperature) had the highest information gain when applied alone (Figure 3). However, the response curve for BIO1 showed no remarkable change in the probability of occurrence across its value range, while the related temperature variable BIO5 (Max temperature of warmest month) exhibited a clear increase in the probability of occurrence as the temperature rises from 20.2 to 30.15 °C (Figure 2, Figure 3, Figure S3, and Figure S4).

The most important unique variable, based on the greatest loss of information when excluded, was DISTANCE for *A. japonicus* and DEM for *D. esakii*. The response curve for *A. japonicus* showed a decreasing probability of occurrence as the distance from wetlands increased. It was observed that the probability of occurrence sharply decreases as elevation increases in the response curve of *D. esakii* for DEM.

In terms of variable contribution, DEM (52.7%) had the highest contribution for *A. japonicus*, followed by DISTANCE (23.9%). Similarly, BIO1 (42.3%) was the most important variable for *D. esakii*, with DEM contributing 24.7% (Table 2). For each species, the top two variables accounted for more than half of the overall contribution. According to the analysis of permutation importance, the top two variables were BIO5 (27.0%) and DEM (26.8%) for *A. japonicus* and BIO15 (30.8%) and BIO5 (29.6%) for *D. esakii*. Overall, elevation (DEM) is the most predictive factor of *A. japonicus* distribution, while annual mean temperature (BIO1) is the most important factor influencing the distribution of *D. esakii*.

**Figure 4:**
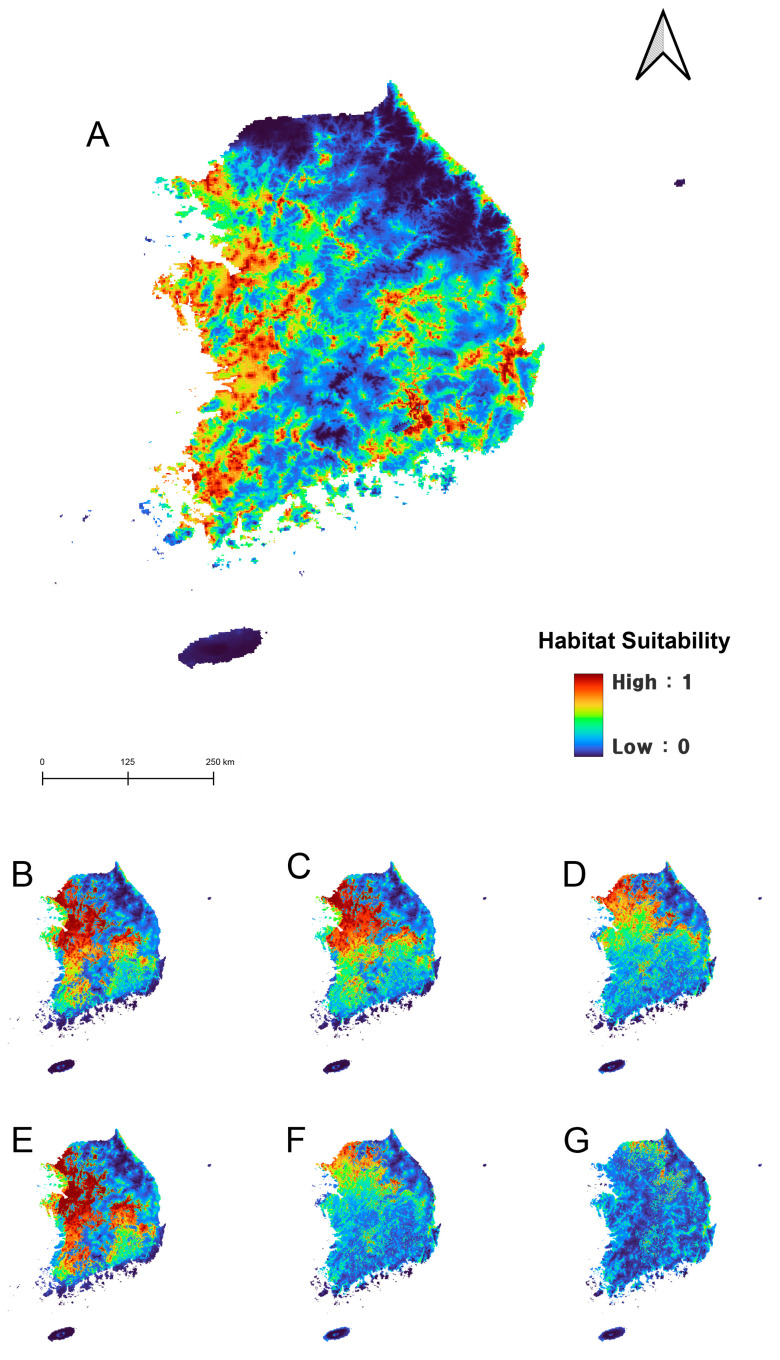
Potential distribution of *Apassus japonicus* in South Korea under the current (1980–2022) climate (**A**), 2055s (2041–2070, **B**–**D**), and 2085s (2071–2100, **E**–**G**). (**B**,**E**), SSP126; (**C**,**F**), SSP370; (**D**,**G**), SSP585.

**Figure 5:**
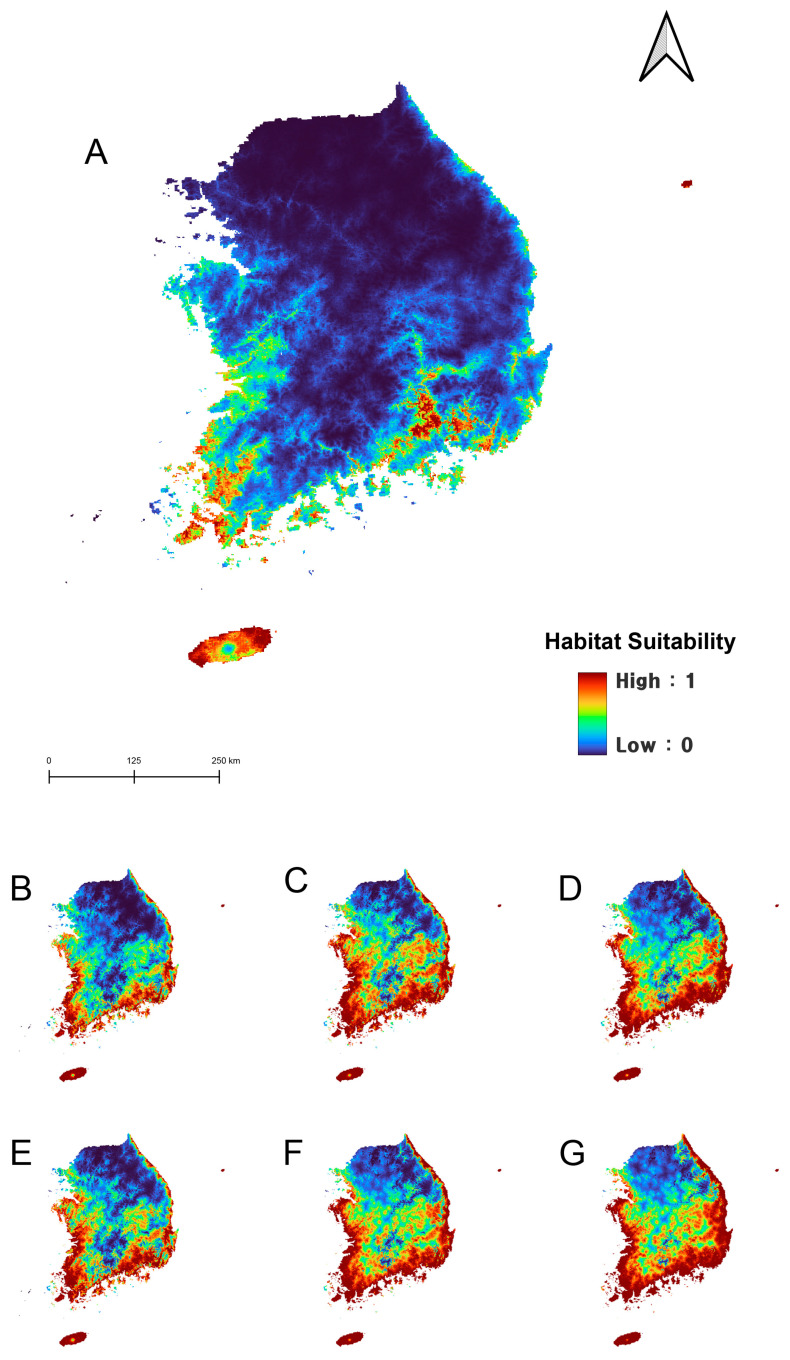
Potential distribution of *Diplonychus esakii* in South Korea under the current (1980–2022) climate (**A**), 2055s (2041–2070, **B**–**D**), and 2085s (2071–2100, **E**–**G**). (**B**,**E**), SSP126; (**C**,**F**), SSP370; (**D**,**G**), SSP585.

Under current climatic conditions, the potential distribution of *A. japonicus* was shown to be widely distributed across South Korea, except in high-altitude regions such as the Taebaek and Sobaek mountain ranges. In contrast, *D. esakii* was expected to be distributed primarily in the southern region of South Korea, with the highest probabilities in Jeju Island and Changnyeong-gun, Gyeongsangnam-do. According to the model, *D. esakii* is more likely along coastal areas rather than inland regions (Figure 4 and Figure 5).

### 3.2. Future Distribution of Two Korean Giant Water Bugs Under Climate Change

The future distribution patterns generated by the MaxEnt model suggest that *A. japonicus* is expected to experience significant range contraction and a northward shift due to climate change (Figure 4). In contrast, *D. esakii* is expected to expand its range northward along the coastal margins of the Korean Peninsula into higher latitudes, without losing its current distribution (Figure 5). Under future climate scenarios, the regions with high distribution probabilities for *A. japonicus* showed a geographical shift, moving from the coastal areas of Jeollanam-do, Jeollabuk-do, Chungcheongnam-do, and Gyeonggi-do toward Gyeonggi-do and Seoul and eventually toward the Gangwon-do region. For *D. esakii*, the high distribution probabilities initially found in Jeju Island, parts of the Jeollanam-do coastline, and Gyeongsangnam-do shifted westward along the coasts of Jeollanam-do and Jeollabuk-do and eastward along the coastlines of Gyeongsangnam-do and Gyeongsangbuk-do, eventually reaching the coastal regions of Chungcheongnam-do, Gyeonggi-do, and Gangwon-do.

**Figure 6:**
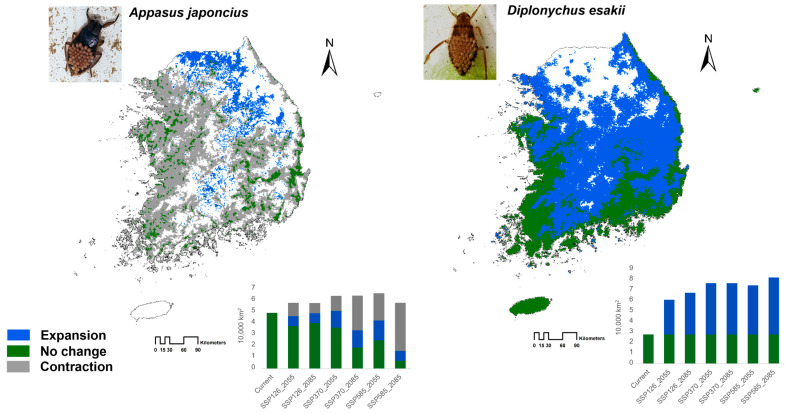
Predicted future distribution of the two giant water bugs, *Appasus japonicus* and *Diplonychus esakii*, under the SSP585 scenario in 2085s.

In the analysis of changes in distribution areas under three different climate change scenarios using binary maps, it was observed that the current distribution of *A. japonicus* could dramatically decrease (9015–42,217 km^2^, 24–86%), depending on the severity of climate change (Table 3). The smallest predicted loss was under SSP126 by the 2085s, with an estimated reduction of approximately 0.9% (including newly expanded areas). Among the scenarios, a net increase in *A. japonicus* distribution was predicted only under SSP370 in the 2055s, with a 3.6% increase compared to its current distribution. Under the other scenarios, the range is expected to shrink, with the most severe reduction predicted under SSP585 by the 2085s, where a net area decreases of 68.1%, and 86.0% of the current range (42,217 km^2^) is predicted to become unsuitable (Figure 6). Overall, due to climate change, the distribution of *A. japonicus* is projected to shift rapidly, with the worst-case scenario leaving its remaining habitat in Gangwon Province, near the Military Demarcation Line—an area currently not well-suited for the species (Figure 6).

Under all future climate scenarios, the distribution of *D. esakii* is expected to expand northward. While maintaining its current distribution area of 27,395 km^2^, it is predicted to gain additional suitable habitat, with its net distribution increasing 2.2 to 2.9 times compared to its current range. The most significant expansion is projected under SSP585, the scenario associated with the highest anticipated temperature increase due to insufficient climate change mitigation. Notably, under the climate change scenarios, habitats currently suitable for *A. japonicus* are expected to become unsuitable in the near future for this species and to become suitable for *D. esakii* (Figure 6). This comparative shift is likely to lead to the occupancy turnover of the two Korean giant water bugs.

## 4. Discussion

Interestingly, although these two species share similar morphology, reproductive behavior, and habitat preferences, they exhibited distinct distribution patterns driven by environmental factors. Both *A. japonicus* and *D. esakii* demonstrated good model performance, with DEM (52.7%) and BIO1 (42.3%) identified as the key factors for each species, respectively. These results reflect the distribution patterns of two Korean giant water bugs in that *A. japonicus* is widely distributed in lowland areas across South Korea, whereas *D. esakii* is more likely to be found in the warmer southern regions (i.e., lower latitudes).

A previous study on *A. japonicus* in Honshu, Japan, indicates its preference for lowland habitats [21], which aligns with our distribution modeling. In both current and future projections, *A. japonicus* is expected to occur in low-elevation habitats including lowland coastal areas. The potential current distribution of *D. esakii* is concentrated in the southern regions, particularly in Changnyeong-gun and Jeju Island, likely due to its preference for warm temperature (e.g., BIO1 and BIO5).

Most species in the genus *Diplonychus*, except for *D. esakii*, are distributed in tropical regions such as the Afrotropical, Oriental, and Australian regions [1]. Although *D. esakii* has been recorded in South Korea, Japan, China, and Taiwan [5], it may actually be endemic to South Korea [10,22,56,57]. Indeed, the *Illustrated Encyclopedia of Fauna & Flora of Korea* [56] noted its distribution as exclusive to South Korea, while *Aquatic Insects of Japan* [57] listed only *D. rusticus*, without including *D. esakii* in the Japanese fauna. Additionally, no published records confirm its presence in Taiwan, and records in China remains uncertain [10]. If this hypothesis proves correct, *D. esakii* would be the only species within the genus *Diplonychus* to inhabit temperate regions. This could explain why *D. esakii* is predominantly distributed in the southern parts of South Korea. Notably, the GPS data we collected confirmed its range extending as far north as N 36° (Taean-gun, Chungcheongnam-do), marking the northernmost limit for both the species and the genus.

The major pattern in the future distribution changes of these two species is a northward shift in their ranges. *Appasus japonicus* is expected to have a higher probability of distribution in the low-elevation western regions of South Korea, with its range shifting from southern to northern areas and from lowland to higher altitudes under climate change (Figure 4). For *D. esakii*, the future distribution is predicted to expand northward without losing parts of its current range, extending from the southern coastal margins further inland to the north (Figure 5), potentially leading to occupancy turnover between the two species.

The two giant water bug species function as top predators in wetland ecosystems, and their range shifts in response to climate change could drive the reassembly of aquatic communities [30,58,59]. Climate change is likely to affect not only these top predators but also other aquatic insects [29], making it increasingly challenging to predict the overall impact on wetland ecosystems in South Korea. Given that most aquatic insects in South Korea have not yet been adequately studied for their responses to climate change [29], the two species of giant water bugs (*A. japonicus* and *D. esakii*), which exhibit both latitudinal and altitudinal range shifts, could serve as valuable indicator species for monitoring and predicting the impacts of climate change on wetland ecosystems in South Korea [60].

Giant water bugs play an important role as top predators in maintaining ecosystem balance [11]; their range shifts could significantly affect lower trophic levels within the food web [8,59,61]. This is an important ecological feature not commonly observed in other wetland insects in South Korea, underscoring the importance of these species as bioindicators in wetland ecosystems. The reassembly of aquatic communities due to climate change can be predicted by monitoring the range shifts of these top predators, and this also helps indirectly assess the responses of other aquatic organisms.

We anticipate that the latitudinal and altitudinal range shifts of the two giant water bug species will also influence other aquatic insects and biodiversity in the ecosystem. Specifically, the observed patterns of habitat contraction and expansion in response to climate change suggest that other temperature-sensitive species may experience similar range shifts [29,30,34]. These findings provide valuable data for understanding species interactions and habitat dynamics in response to climate change within the wetlands of South Korea. Thus, giant water bugs may be considered useful species for understanding the aquatic insect community in South Korea, and their role is likely to become increasingly important in developing strategies for conserving wetland biodiversity in response to future climate change.

Our distribution models should be interpreted cautiously. Contrary to expectations, land cover did not significantly contribute to the models for both species. This may be due to the resolution of the land cover data, which failed to capture the fine-scale habitats (often within tens or even one meter) of giant water bugs. For instance, small habitats such as artificial ponds in urban areas, agricultural paddy fields, and small water bodies like irrigation ponds (*doombeong*) are sometimes key microhabitats for these species [62] but were not classified under categories of the wetland (5) or water (7). *Appasus japonicus* has a broader distribution than *D. esakii*, with it being frequently found in both lentic waters and slow-flowing riverine wetlands. *Diplonychus esakii*, in contrast, does not prefer riverine wetlands and typically favors lentic environments [63]. Neither species is strictly tied to natural or artificial habitats, but both require environments with suitable vegetation, food resources, and water quality [64]. Microhabitat conditions like these are also important factors influencing the distribution of giant water bugs, but they could not be fully accounted for in this study. Furthermore, genetic attributes related to adaptive capacity to environmental changes [30,65,66] were not considered in this study. *Diplonychus esakii* is, in fact, a species of conservation concern due to its low level of genetic diversity and limited gene flow between populations [67], which may make it more vulnerable to climate change. This could result in a weaker or slower range expansion than predicted by our model, which is based solely on environmental variables.

Wetland ecosystems provide critical functions such as climate regulation and carbon sequestration [32]. Wetland degradation not only directly reduces the available habitat for these species but also limits the capacity they need to adapt to climate change. Consequently, wetland conservation plays a crucial role in enhancing the resilience of these species and ensuring their long-term survival. In South Korea, the National Institute of Ecology conducts nationwide inland wetland surveys, and the Ministry of Oceans and Fisheries oversees coastal wetland surveys [31,68,69]. However, due to the lack of management measures and oversight for non-designated wetlands, there is a risk of wetland degradation or reclamation [31]. Following the four strategies outlined in the Wetland Conservation Plan [35]—scientific-based wetland surveys, effective wetland conservation and management, sustainable wetland use, and strengthening cooperation for wetland management—we propose continued monitoring and management of the core habitats of these two giant water bug species.

## 5. Conclusions

In this study, we developed species distribution models (SDMs) based on ten environmental variables to predict both the current and future distributions of two Korean giant water bug species, *Appasus japonicus* and *Diplonychus esakii*, for the first time. Under climate change scenarios, the distribution of the two species is projected to shift toward higher latitudes compared to the current distribution. The predicted distributional changes suggest that *D. esakii* will occupy the range vacated by *A. japonicus*. These results provide valuable insights into the environmental factors influencing the distribution of the two Korean giant water bugs and their potential range shifts under climate change. These findings offer essential baseline information for wetland conservation efforts. Future research should incorporate a broader range of environmental variables with biological variables and implement long-term monitoring to better understand the impacts of climate change on aquatic insects and wetland ecosystems.

## Figures and Tables

**Table 1:** List of environmental variables for the study. Bold indicates the selected variables.

Variable	Definition	Unit	Source
**BIO1**	**Annual mean temperature**	**°C**	[43]
**BIO2**	**Mean diurnal range (max temp–min temp)**	**°C**	″
BIO3	Isothermality (BIO2/BIO7) × 100	%	″
BIO4	Temperature seasonality (standard deviation × 100)		″
**BIO5**	**Maximum temperature of warmest month**	**°C**	″
BIO6	Minimum temperature of coldest month	°C	″
BIO7	Temperature annual range (BIO5—BIO6)	°C	″
BIO8	Mean temperature of wettest quarter	°C	″
BIO9	Mean temperature of driest quarter	°C	″
BIO10	Mean temperature of warmest quarter	°C	″
BIO11	Mean temperature of coldest quarter	°C	″
**BIO12**	**Annual precipitation**	**mm**	″
**BIO13**	**Precipitation of wettest month**	**mm**	″
**BIO14**	**Precipitation of driest month**	**mm**	″
**BIO15**	**Precipitation seasonality (coefficient of variation)**		″
BIO16	Precipitation of wettest quarter	mm	″
BIO17	Precipitation of driest quarter	mm	″
BIO18	Precipitation of warmest quarter	mm	″
BIO19	Precipitation of coldest quarter	mm	″
**DEM**	**Surface elevation above sea level**	**m**	″
**DISTANCE**	**Distance from wetland**	**km**	[44]
**LANDCOVER**	**Land cover**	**Categorical**	[45]

**Table 2:** The contribution (%) and permutation importance (%) of the variables in the MaxEnt model for two giant water bug species.

Species	*Appasus japonicus*		*Diplonychus esakii*	
Variable	Contribution (%)	Permutation Importance (%)	Contribution (%)	Permutation Importance (%)
BIO1	4.1	4.8	42.3	0.2
BIO2	2.2	4.0	9.7	17.5
BIO5	6.9	27.0	3.8	29.6
BIO12	1.9	7.6	0.9	3.3
BIO13	2.3	5.4	0.5	0.5
BIO14	1.2	1.7	1.6	1.3
BIO15	2.9	10.5	11.1	30.8
DEM	52.7	26.8	24.7	8.7
DISTANCE	23.9	11.8	3.3	6.3
LANDCOVER	1.9	0.5	2.2	1.7

**Table 3:** Predicted future distribution area of the two giant water bugs, *Appasus japonicus* and *Diplonychus esakii*. Unit: square kilometers.

Species/Responses	Distribution Changes Under Different Climate Change Scenarios and Periods						
	Current	SSP126_2055s	SSP126_2085s	SSP370_2055s	SSP370_2085s	SSP585_2055s	SSP585_2085s
*Apaasus japonicus*							
Range expansion		8839	8581	14,906	15,241	17,363	8767
No change	49,104	37,316	40,089	35,951	18,453	24,885	6887
Range contraction		11,788	9015	13,153	30,651	24,219	42,217
*Diplonychus esakii*							
Range expansion		32,558	39,252	48,236	48,172	46,167	53,462
No change	27,395	27,395	27,395	27,395	27,395	27,395	27,395
Range contraction		-	-	-	-	-	-

## Data Availability

Data are available from the authors upon request.

## Supplementary Materials

The following supporting information can be downloaded at: https://www.mdpi.com/article/10.3390/insects15100820/s1. Figure S1: Pearson’s correlation coefficients (r) of the 19 bioclimatic variables in South Korea; Figure S2: The evaluation values of the parameter combinations, implemented in ENMeval. *Appasus japonicus* (A,B) and *Diplonychus esakii* (C,D); Figure S3: Area under the receiver operating curve (AUC), jackknife test, and response curves of variables of the optimal MaxEnt model for *Appasus japonicus* (Figure 2); Figure S4: Area under the receiver operating curve (AUC), jackknife test, and response curves of variables of the optimal MaxEnt model for *Diplonychus esakii* (Figure 3); Table S1: The 671 rarefied occurrence records of *Appasus japonicus* within 1 km; Table S2: The 154 rarefied occurrence records of *Diplonychus esakii* within 1 km.
