# Supplementary figures and images for: The Effect of Climate Change on Indicator Wetland Insects: Predicting the Current and Future Distribution of Two Giant Water Bugs (Hemiptera: Belostomatidae) in South Korea
Source: Insects. 2024 Oct 19;15(10):820. doi: 10.3390/insects15100820 (PMC11508284; doi:10.3390/insects15100820)

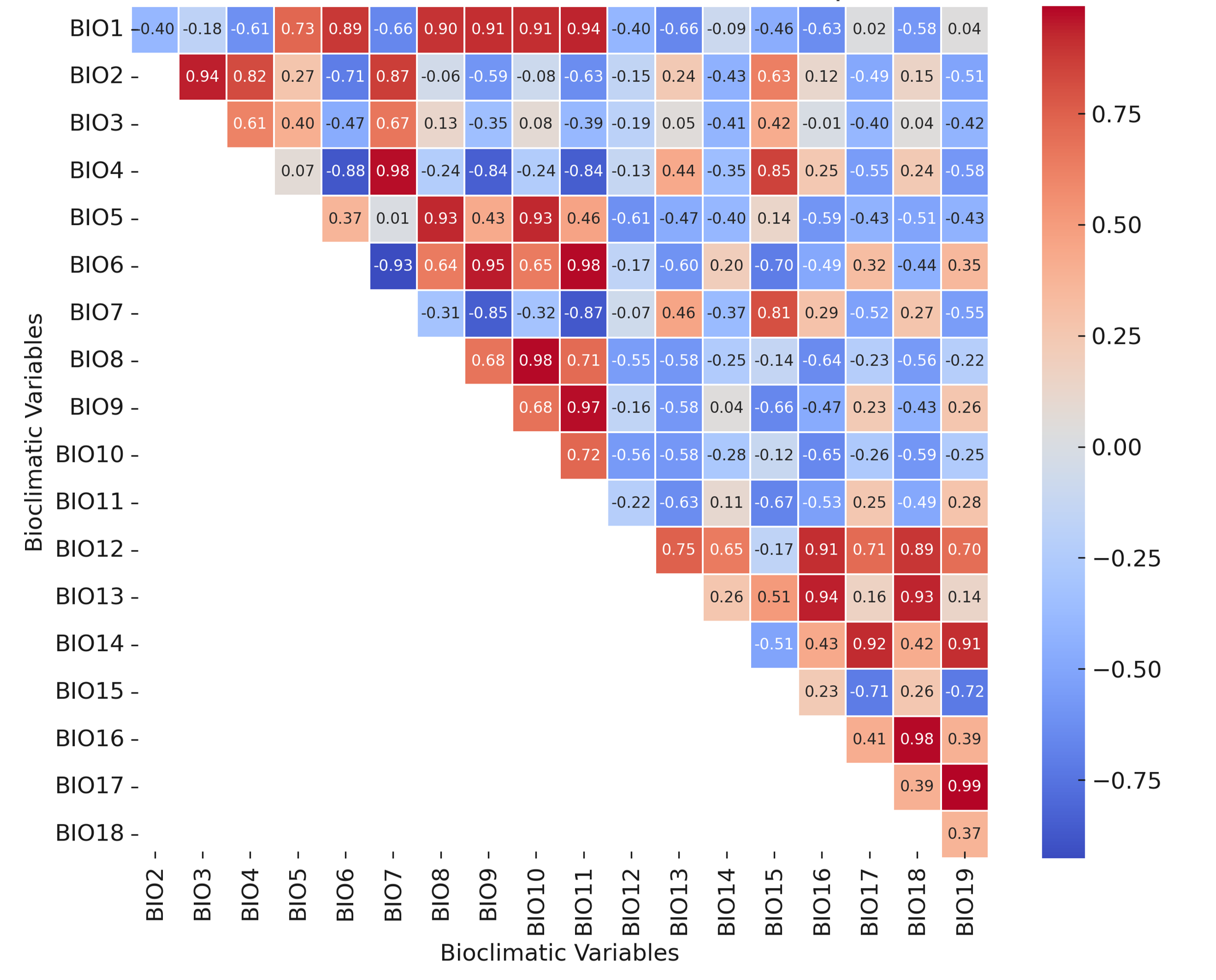

Supplement: Supplementary file 1 [file insects-15-00820-s001.zip › Figure_S1.tif]

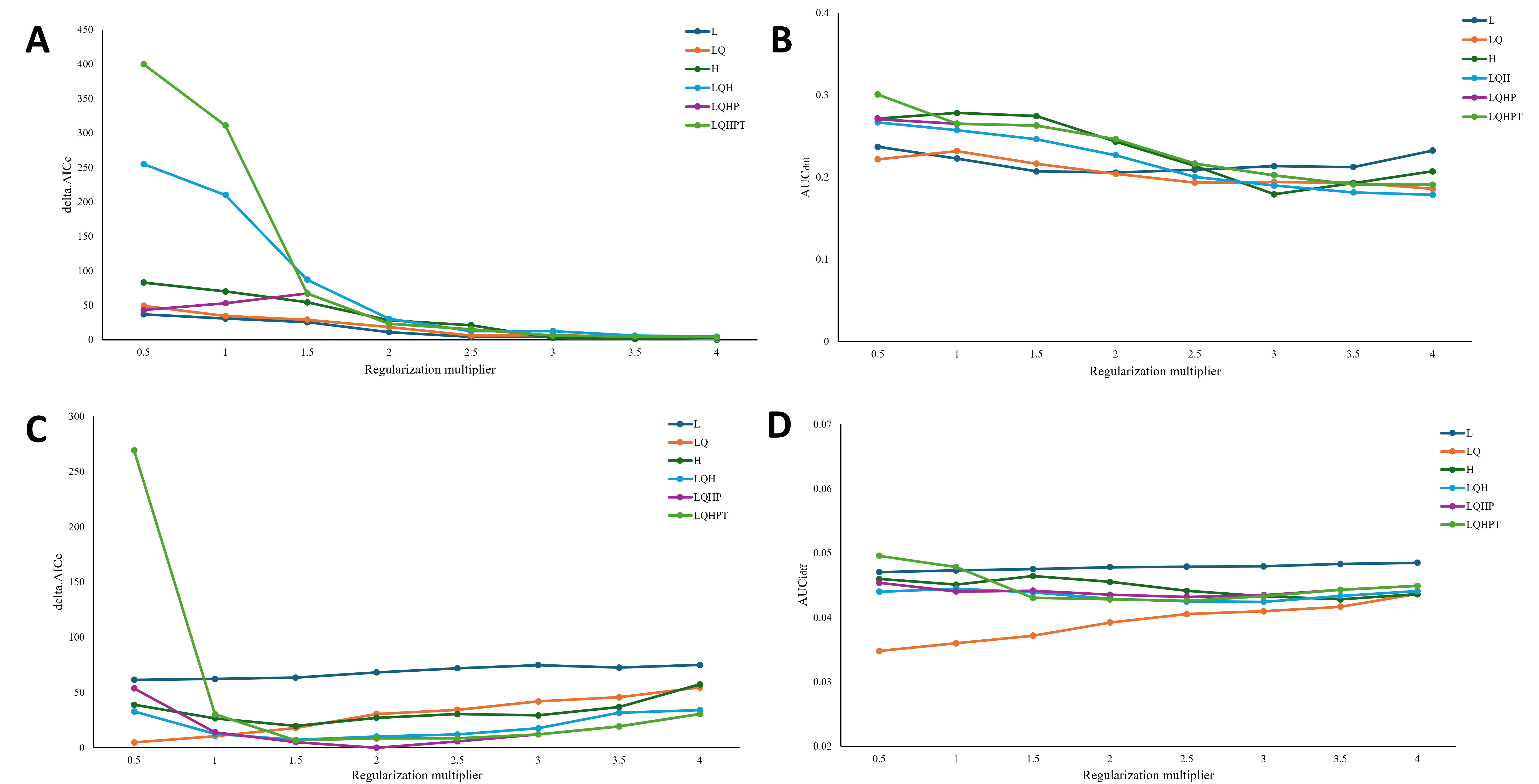

Supplement: Supplementary file 1 [file insects-15-00820-s001.zip › Figure_S2.tif]

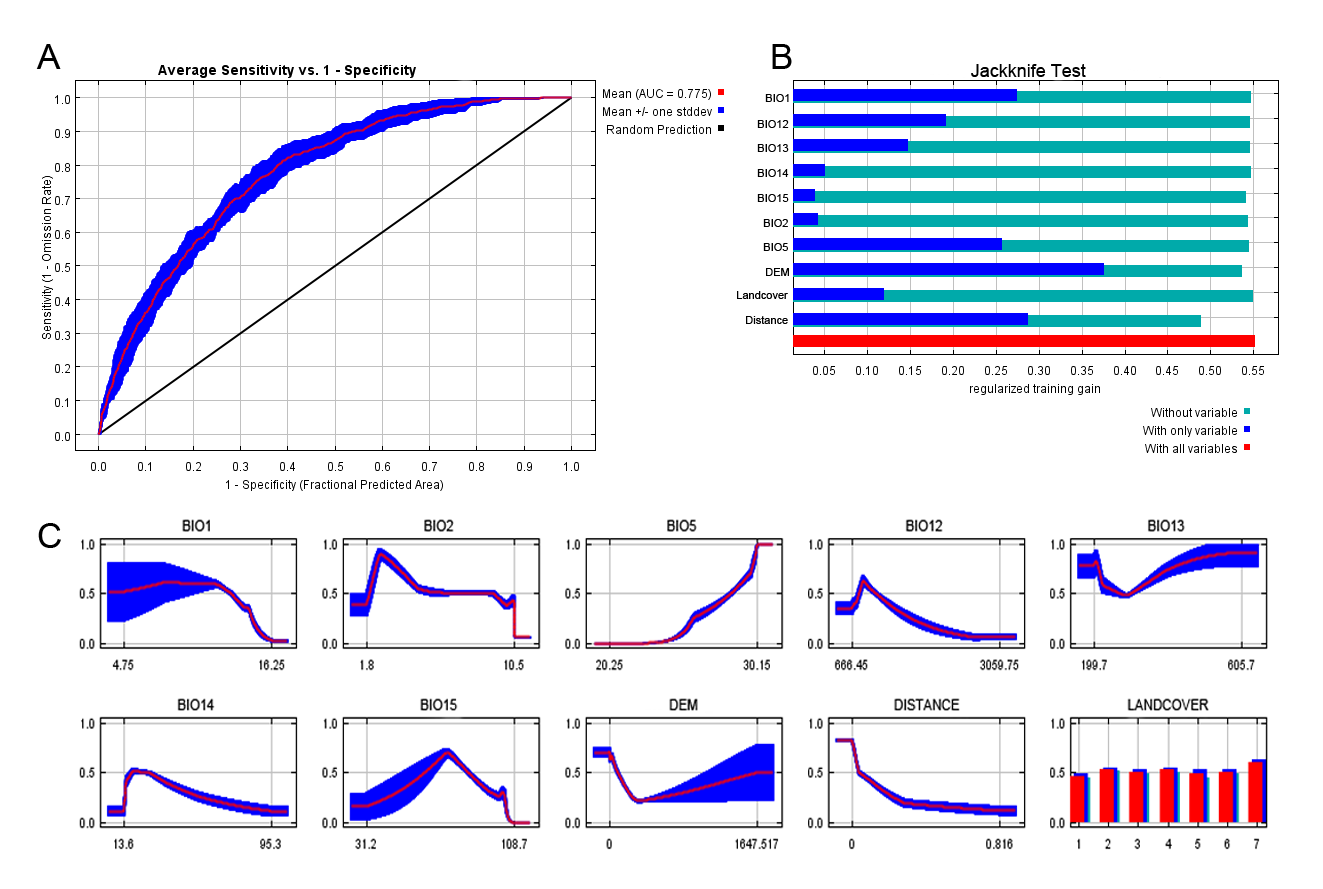

Supplement: Supplementary file 1 [file insects-15-00820-s001.zip › Figure_S3.png]

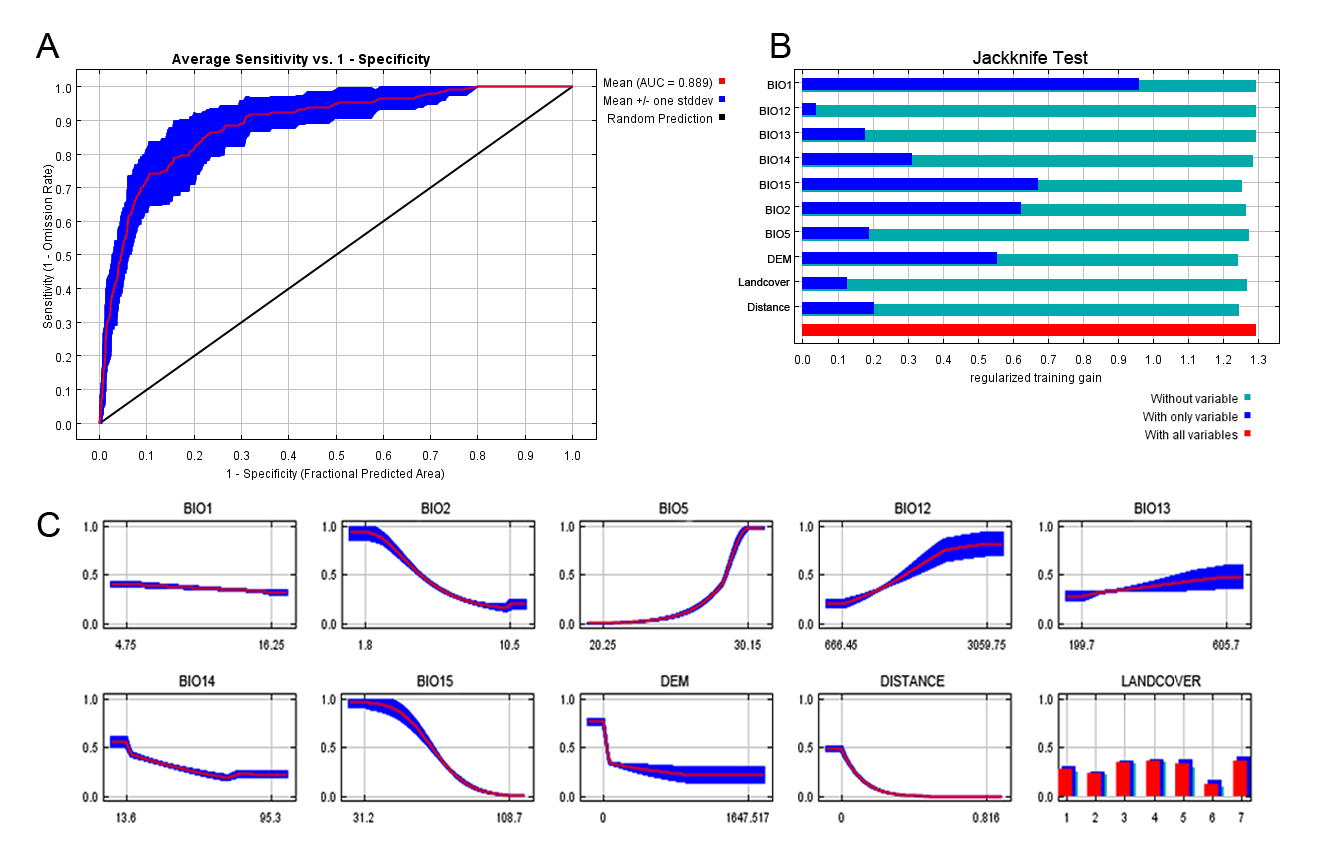

Supplement: Supplementary file 1 [file insects-15-00820-s001.zip › Figure_S4.png]
